# Anti-VEGF Cancer Therapy in Nephrology Practice

**DOI:** 10.1155/2014/143426

**Published:** 2014-08-24

**Authors:** Hassan Izzedine

**Affiliations:** ^1^Department of Nephrology, Pitie-Salpetriere Hospital, 75013 Paris, France; ^2^Department of Nephrology, Monceau Park International Clinic, 75017 Paris, France

## Abstract

Expanded clinical experience with the antivascular endothelial growth factor (VEGF) agents has come with increasing recognition of their renal adverse effects. Although renal histology is rarely sought in antiangiogenic-treated cancer patients, kidney damage related to anti-VEGF is now established. Its manifestations include hypertension, proteinuria, and mainly glomerular thrombotic microangiopathy. Then, in nephrology practice, should we continue to perform kidney biopsy, and what should be done with the anti-VEGF agents in case of renal toxicity?

## 1. Introduction

Angiogenesis is a vital physiologic process needed for growth and development [1, 2]. In the renal glomeruli, podocytes express vascular endothelial growth factor (VEGF), whereas VEGF receptor tyrosine kinases are expressed by both podocytes and glomerular endothelial cells [3]. The biological functions of VEGF are mediated by its binding to one of the VEGF receptor tyrosine kinases, which include VEGFR-1 (Flt-1), VEGFR-2 (KDR/Flk-1), and VEGFR-3 (Flt-4). A major regulator of angiogenesis is VEGF and its cognate receptor VEGFR2. Antiangiogenesis agents are among the most commonly used anticancer agents in oncology practice today. Therapeutic approaches target the VEGF ligand (bevacizumab (anti-VEGF monoclonal antibody), aflibercept (VEGF Trap)) or the tyrosine kinase receptor [sunitinib, sorafenib, and pazopanib] TKI interfere with the activity of VEGFR and other growth factors, among them PDGF receptors (PDGFRs), stem cell factor receptor (c-kit), FMS-like tyrosine kinase-3 (Flt-3), and b-raf and Bcl-Abl. They are, thus, commonly named as multitargeted TKI.  Table 1 summarized several selected FDA approved targeted antiangiogenic agents.

## 2. Renal Adverse Effects

The filtration barrier of the renal glomeruli is formed by endothelial cells (ECs), podocytes, and basement membrane components. VEGF, which is expressed by podocytes both during development and in the adult, activates VEGFR-2 on glomerular capillary endothelial cells. Interaction of VEGF produced by podocytes with VEGFR2 on glomerular ECs is critical to the normal function and repair of the system. Clinically, renal adverse effects following anti-VEGF therapies may present as hypertension, asymptomatic proteinuria, and, rarely, nephrotic syndrome or acute renal failure. The underlying pathological changes are not always clear. In the few cases where renal biopsies were performed, pathological findings have included proliferative glomerulopathies, thrombotic microangiopathy [4], and, rarely, interstitial nephritis [5]. In preclinical murine models, heterozygous deletion of* VEGF* in podocytes led to loss of EC fenestration, loss of podocytes, mesangiolysis, and proteinuria [6, 7] suggesting that VEGF have a critical protective role in the pathogenesis of microangiopathic process [8].

### 2.1. Hypertension

Hypertension is one of the best-documented and most frequently observed AE of VEGF/VEGFr inhibitors [9–16]. It is a VEGF inhibitor class dependent, dose-dependent, and additive adverse event [11]. Hypertension can occur any time after the initiation of treatment and may be involved after prolonged treatment. This side effect usually can be managed with oral antihypertensive agents, and anti-VEGF treatment can be continued without reduction in dose. The effect of anti-VEGF agents on blood pressure is dose-dependent and the extent of hypertension might reflect the extent of target inhibition. In a phase 2 study in patients with renal-cell carcinoma (RCC) treated with either placebo, 3 mg/kg bevacizumab, or 10 mg/kg bevacizumab, the rate of hypertension was significantly higher in the high-dose group (36%) compared with the low dose group (3%) [17]. With small-molecule VEGFr TKis, the increment rise in blood pressure was also proportional to dose [18]. More-specific and potent VEGFr TKIs, such as cediranib and axitinib, are associated with a higher rate of hypertension compared to sunitinib or sorafenib at the MTD [19]. Because blood pressure is a known on-target effect for anti-VEGF agents, blood pressure is a potential pharmacodynamic marker for anti-VEGF therapy. In a retrospective analysis of sunitinib in 40 patients with cytokine-refractory RCC, only hypertension, particularly grade 3, was associated with a higher treatment response rate [20]. A similar finding was demonstrated in a prospective study of 43 patients with metastatic RCC treated with bevacizumab. In that study, a significantly longer median time to progression was observed for patients with hypertension than for patients with BP <150/100 mmHg (8.1 versus 4.2; *P* = .036) [21]. Ravaud and Sire [22] evaluated hypertension and efficacy in 93 patients receiving either sunitinib, sorafenib, or bevacizumab as first-, second-, or third-line therapy. Among the eligible patients with grade ≥2 hypertension, 88% had a clinical benefit (defined as an objective response or stable disease) and 53% benefited for ≥6 months, versus 55% and 35%, respectively. More recently, the predictive power of hypertension was evaluated in a retrospective analysis of the phase III CALGB 90206 study, which demonstrated that patients on bevacizumab plus interferon who developed grade ≥2 hypertension had significantly greater progression-free survival and overall survival times than patients who did not develop hypertension [23]. For this reason, there have been several reports correlating treatment related blood pressure changes with clinical outcome [20, 21, 24–26]. However, one analysis used patient-specific data including individual blood pressure values from eight phase III controlled trials with bevacizumab conducted by Genentech or Roche [27] found that treatment-related hypertension did not predict benefit from bevacizumab. Prospective trials are needed to clarify this issue.

**Figure 1:**
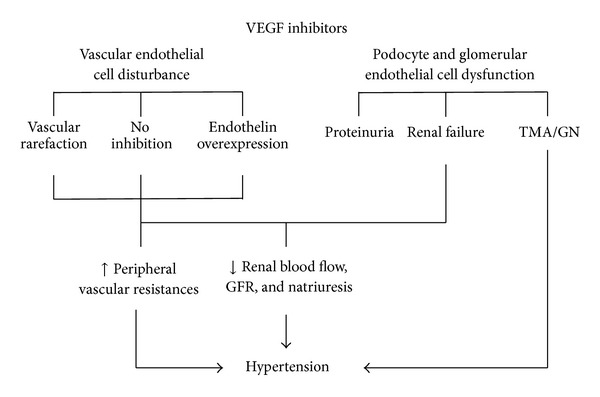
Potential mechanisms of hypertension related to anti-VEGF agents.

VEGFr2 signaling generates nitric oxide and prostaglandin, which induces EC-dependent vasodilatation in arterioles and venules [28, 29] the component of vasculature that has most impact on blood pressure. Hence, blockage of VEGF would lead to vasoconstriction [29–31]. Vascular rarefaction has also been hypothesized as a mechanism of hypertension induced by anti-VEGF therapy [32]. Hypertension may also reflect a renal parenchymal disorder (i.e., acute renal injury, glomerulopathy, and thrombotic microangiopathy) (Figure 1).

Furthermore, many factors, including preexisting hypertension, cancer type, VEGF polymorphism, chemotherapy and its side effects, other medications, and activity and diet may play a role. Patients with preexisting hypertension are generally more likely to develop further elevation in blood pressure when receiving anti-VEGF therapy. The risk of hypertension related to anti-VEGF therapy is also higher in patients with metastatic RCC compared to other indications as reported in sorafenib (17% and 5% of RCC [33] and hepatocellular carcinoma [34] treated patients the same dose of sorafenib, resp.) and sunitinib [35, 36] phase 3 trials. Certain* VEGF* polymorphisms might be associated with a lower risk of grade 3 or 4 hypertension in bevacizumab-treated breast cancer patients [37] and under sunitinib therapy [38].

Hypertension is a known risk for more severe complications, such as reversible posterior leukoencephalopathy syndrome. RPLS is attributed to hypertensive encephalopathy and endothelial dysfunction leading to breakdown of the blood-brain barrier, focal cerebral oedema, or vasospasm. RPLS is a serious but reversible condition characterised by onset of headache, altered mental function, seizures, visual impairment or blindness, and occipital-parietal subcortical cerebral oedema evident by computed tomography and magnetic resonance imaging. RPLS has been reported in patients on bevacizumab [39], sunitinib, or sorafenib [40].

In patients with cancer, the primary goal of hypertension management is to maintain an acceptable blood pressure level to allow safe delivery of antiangiogenesis therapy. In order to prevent life-threatening complications, while minimizing delay and/or dose attenuation of anticancer therapy, close monitoring of blood pressure and timely initiation or titration of hypertension medications are critical. The Joint National Committee on Prevention, Detection, Evaluation, and Treatment of High Blood Pressure (JNC7) stipulate that target blood pressure control should be <140/90 mmHg in the general population and <120/80 mmHg in patients with diabetes or renal dysfunction [41]. Although this ideal blood pressure target does not need be reached to allow continuation of antiangiogenesis therapies, given the effectiveness of hypertensive medication, this goal should be achievable in most patients. Hypertension can be controlled with standard oral hypertensive medications in most cases where therapeutic doses of these anti-VEGF agents are used. In patients who develop hypertensive crisis, permanent discontinuation of anti-VEGF therapy is recommended.

### 2.2. Proteinuria

As for hypertension, proteinuria is a VEGF inhibitor class dependent, dose-dependent, and additive adverse event [11]. Proteinuria was found in 23% of 1132 patients in clinical trials of bevacizumab in various types of cancer and was more common in patients receiving bevacizumab plus chemotherapy than in patients on chemotherapy alone [12, 13]. Significant increase in urine protein (grade 3, >3.5 g protein per 24 h urine) is less common, occurring in 3% of patients in most clinical trials [42–45] and in up to 7-8% of patients with RCC [17, 46]. In rare cases, patients with asymptomatic proteinuria can progress to nephrotic syndrome (<0.5% of patients) [47]. In a follow-up review of more than 12,000 patients, Zhu et al. identified the incidence of high-grade proteinuria (grade 3 or worse) at 2.2% with a relative risk (RR) of 4.79 (95% CI: 2.71–8.46). The RR of developing nephrotic syndrome with chemotherapy containing bevacizumab (when compared with chemotherapy without bevacizumab) was 7.78 [48]. Proteinuria is typically asymptomatic and decreases after treatment ends. Proteinuria is rarely reported in clinical trials with sunitinib or sorafenib, although how closely patients were monitored for this adverse effect is unclear. With axitinib, a potent and specific VEGFr TKi, 32% of patients (17 of 52) with RCC developed grade 2 or higher proteinuria (as measured by a dipstick) and a few patients had proteinuria >1 g per 24 h urine [49]. The common occurrence of proteinuria after inhibition of VEGF signalling reflects the importance of VEGF in normal renal function [6, 7]. Targeted heterozygous deletion of VEGF in podocytes results in renal pathology manifested by loss of endothelial fenestrations in glomerular capillaries, proliferation of glomerular endothelial cells (endotheliosis), loss of podocytes, and proteinuria in mice [6, 7]. Pharmacological inhibition of VEGF signalling in mice also reduces endothelial fenestrations in glomerular capillaries [50]. Inhibition of VEGF-dependent interactions between podocytes and glomerular endothelial cells disrupts the filtration barrier, which in turn leads to dose-dependent proteinuria [6, 50]. Patients treated with anti-VEGF agent should be monitored for proteinuria, by either dipstick or calculation of the urine protein/creatinine ratio on spot urine samples. Anti-VEGF agents should be interrupted if 24 h urine protein exceeds 2.0 or 3.5 g, and these agents should be permanently discontinued upon development of nephrotic syndrome. Serious impairment of renal function is rare. Indeed, in clinical practice, oncologists and nephrologists usually manage proteinuria related to anti VEGF treatment only when at nephrotic range or when associated with renal insufficiency. However, we found that proteinuria induced by anti VEGF therapy, even if weak and without associated renal insufficiency, may reflect a renal TMA in 35% of cases [51]. Hence, proteinuria, even if weak and without associated renal insufficiency, may reflect a serious histological renal disease.

### 2.3. Renal Thrombotic Microangiopathy

Thrombotic microangiopathy (TMA) has been described in biopsy samples from case reports of patients treated with bevacizumab [8, 52, 53], VEGF-Trap [54], and sunitinib [55–57]. TMA associated with VEGF/VEGFr inhibitors was mostly localized to the kidney, and systemic manifestations (e.g., thrombocytopenia or schistocytosis) were present only in half of these patients [58]. Available data indicate that systemically evident TMA is very rare with anti-VEGF therapies. However, the use of more than one anti-VEGF agent in combination might enhance the risk. In a phase 1 dose escalation trial of concurrent bevacizumab (10 mg/kg every 2 weeks) and escalating doses of sunitinib (25 mg, 37.5 mg, or 50 mg daily for 4 out of 6 weeks) in patients with RCC, 5 of the 12 patients at the highest dose level developed systemic TMA, or microangiopathic haemolytic anemia; clinical presentations in these cases included thrombocytopenia, schistocytes, hypertension, and varying degrees of proteinuria [11, 59].

## 3. In Nephrology Clinical Practice

### 3.1. Comparing the Anti-VEGF Agents: Are There (Renal) Toxicity Differences?

Anti-VEGF treatments in general have been relatively well tolerated when compared with traditional chemotherapy. This may relate to the tumor specificity of VEGF expression and/or the redundancy of angiogenesis in the host. Common toxicities thought to be related to on-target effects include fatigue, hypertension [60–62], proteinuria, delayed wound healing, and chemical hypothyroidism (often without clinical symptoms) [63–66]. Several rare side effects have also been reported in multiple trials and include bleeding and/or thrombosis (which can be severe or fatal), intestinal and nasal septal perforation [67], effects on growth plates [68], and posterior reversible encephalopathy syndrome (PRES), also known as reversible posterior leukoencephalopathy syndrome (RPLS) [69].

The differences in binding and complex formation between VEGF ligand targeting agents bevacizumab and aflibercept could have important implications in terms of the AE profile, for example, in terms of renal damage and proteinuria resulting from the deposition of VEGF-A-bevacizumab complexes in the kidney [70, 71]. Indeed, unlike bevacizumab VEGF, aflibercept formed stable complexes in the circulation that remained bound to VEGF-A. In addition, although aflibercept formed inert 1 : 1 complexes with VEGF-A, bevacizumab formed heterogeneous multimeric immune complexes that were rapidly cleared from the circulation [71]. Many of TKI agents have unwanted “off-target” AEs associated with their inhibition of non-VEGFR kinases compared with VEGF ligand targeting agents [72, 73]. These off-target AEs included fatigue, diarrhea, nausea, anorexia, and hand-foot reaction [73, 74]. In the past 7 years, we have managed 78 patients who developed biopsy-proven kidney disease under anti VEGF therapy. Those patients were referred for proteinuria, hypertension, and/or renal insufficiency after the initiation of anti VEGF therapy. Of those patients, 65.4% (51 pts) experienced renal thrombotic microangiopathy (TMA) and twenty-seven patients (34.6%) had variable glomerulopathies mainly minimal change disease and/or focal segmental glomerulosclerosis (MCN/FSGS) sometimes in collapsing variant [58]. We found that MCN/FSGS-like lesions developed mainly under TKIs, whereas TMA complicated anti-VEGF ligand [58]. Immunomorphological and molecular studies suggest that RelA and c-mip define two separate glomerular damages associated with anti-angiogenic drugs, based on two distinct pathophysiological mechanisms. Indeed, we show that MCN/FSGS lesions are associated with high abundance of c-mip. In contrast, in TMA resulting from anti-VEGF therapy, c-mip is not detected, while RelA is produced at high levels by podocytes and glomerular endothelial cells [58].

### 3.2. Kidney Biopsy: Why Is It Done?

Proteinuria after inhibition of VEGF signalling will frequently and promptly disappear upon stopping the responsible agent and achieving blood pressure control, and rarely acute renal failure can develop. Furthermore, bleeding is one of the most severe and potentially life-threatening toxicities of antiangiogenic drugs, particularly bevacizumab which retains the highest frequency. Hence, renal biopsy is rarely performed in patients with proteinuria or renal insufficiency under VEGF targeted therapies with the result of an unassessable true rate of glomerulopathy or renal-localized TMA. Therefore, should we reserve the renal biopsy only for research? In my personal view, we must continue to make kidney biopsy in clinical practice for the following reasons: (a) half and 100% of TMA under anti-VEGF are exclusive renal-localized clinically and histologically, respectively [58], (b) proteinuria induced by anti-VEGF therapy, even if weakly and without associated renal insufficiency, may reflect a serious histological renal disease (35% of our 78 TMA patients had proteinuria less than 1 gram per 24 h) [51], and (c) proteinuria may be related to a paraneoplastic membranous nephropathy (2 unpublished personal cases) requiring instead a therapeutic strengthening rather than stopping the anti-VEGF. Moreover, to minimize the hemorrhagic risk, the biopsy should be performed by an interventional nephrologist and/or by transjugular way.

### 3.3. Once Kidney Disease Related to Anti-VEGF Is Diagnosed, Do We Continue, Discontinue, or Change the Treatment?

In clinical practice, the decision to continue, discontinue, or change a treatment is a daily problem. “When to stop” may be interpreted in 2 ways: either the temporary suspension of anti-VEGF agents without any loss of benefit or a final decision to stop. In many cases, this decision depends strongly on the interpretation of the outcome change from baseline. When the change in outcome indicates effectiveness, continuing the treatment is a logical decision. Similarly, discontinuing the treatment is appropriate when it has not been effective. Often, the problem is whether we stop or not an effective treatment due to its renal side effects.

I think we should distinguish two groups of patients: those with glomerular disease type MCN/FSGS for which antihypertensive and antiproteinuric treatments can stabilize kidney disease and those with renal TMA. There are only few published data on renal outcome in this setting. In one case of sunitinib induced renal TMA, blood pressure and renal function remained stable and proteinuria became undetectable under irbesartan over 3 months while sunitinib was continued [55]. Another patient who developed TMA under bevacizumab had favourable response after stopping bevacizumab (normalising blood pressure, disappearance of haemolysis, and return of renal function to previous baseline level). Sunitinib, introduced 2 months later, was stopped after 3 weeks of treatment as a result of the recurrence of a severe TMA. Once again, the response of this second episode was favourable in the days after stoppage of sunitinib, although ten courses of plasma exchange were initially needed [52]. In my own experience, four patients required maintains of an anti-VEGF treatment despite renal TMA. One patient who experienced TMA under VEGF Trap was switched to bevacizumab displaying an absence of proteinuria and stable renal function two years later without TMA recurrence. Two patients with TMA related to bevacizumab continued this therapy in association with antihypertensive drugs for 8 months despite persistent proteinuria and the occurrence of systemic manifestations (such as hemolysis, thrombocytopenia, and schistocytosis) but renal function remained stable. For the last patient, the reintroduction of bevacizumab resulted in a more severe recurrence of TMA (hematological and renal signs). It, therefore, seems more reasonable to stop the culprit drug in case of TMA. In case the offender treatment is the only active one, a temporary halt is still necessary time to obtain an optimal blockade of the renin angiotensin aldosterone system before its reintroduction at half dose if possible and to adjust the dosage according to efficacy and clinical tolerance. Treatment reintroduction or continuation must meet two requirements: a rigorous and necessary monitoring of renal and hematological parameters and discontinuation of treatment in case of recurrence of TMA. Careful risk-benefit assessment for individual patients is important and should take into account risk factors related to the host and the tumour.

In conclusion, anti-VEGF agents may induce hypertension proteinuria and TMA related to endothelial cell dysfunction and regression of fenestrated capillaries. At the current time, approaches to toxicity management and treatment modifications are largely empirical. Therapeutic or observational studies are needed to identify baseline risk factors and early signs of serious AEs and collect data on safety if antiangiogenesis agents be resumed after recovery from AEs.

## Figures and Tables

**Table 1:** Selected FDA approved targeted anticancer drugs.

Generic (trade) names	Target gene or receptor	Indication
IV antiangiogenic drugs		
Bevacizumab (Avastin)	VEGF-A	Metastatic colorectal cancer (mCRC) (with chemotherapy) Metastatic NSCLC (with chemotherapy) Metastatic breast cancer (with chemotherapy) Recurrent glioblastoma (monotherapy) Metastatic renal cell carcinoma (RCC) (with IFN-a)
VEGF Trap (Aflibercept)	VEGF A, PIGF	mCRC (second-line)
Temsirolimus (Toricel)	mTOR	Advanced RCC

Oral antiangiogenic drugs		
Dasatinib (Syrcell)	BCR-ABL	Philadelphia chromosome-positive (Ph+) CML
Imatinib (Gleevec)	BCR-ABL	Ph+ CML; gastrointestinal stromal tumor (GIST)
Nilotinib (Tasigna)	BCR-ABL	Ph+ CML
Bosutinib (Bosulif)	BCR-ABL, Src	Ph+ CML
Ponatinib (Iclusig)	BCR-ABL	ALL and CML
Vemurafenib (Zelboraf)	BRAFV600E	Melanoma
Vismodegib (Erivedge)	SMO	Basal cell carcinoma
Ruxolitinib (Jakafi)	JAK1/2	Myelofibrosis
Gefinitib (Iressa)	EGFR	NSCLC
Erlotinib (Tarceva)	EGFR	NSCLC and pancreatic cancer
Crizotinib (Xalkori)	EML4-ALK	NSCLC
Abiraterone (Zytiga)	CYP17A1	Prostate cancer
Enzalutamide (Xtandi)	AR	Prostate cancer
Regorafenib (Stivarga)	VEGFR2, PDGFR, FGFRs, Tie2, RAF-1, BRAF, BRAFV600E, Abl	Metastatic colorectal cancer (refractory disease)
Lenalidomide (Revlimid)	Anti-tumor, immunomodulatory	Multiple myeloma
Lapatinib (Tykreb)	EGFR, HER2/neu	Breast cancer
Sunitinib (Sutent)	VEGFRs, PDGFR, VEGF, cKIT, RET, CSF-1R, flt3	GIST; advanced RCC; Unresectable locally advanced or metastatic pancreatic neuroendocrine tumours
Sorafenib (Nexavar)	VEGFR, PDGFR, C-Raf, B-Raf, MAP Kinase, cKIT	Advanced RCC Unresectable hepatocellular carcinoma
Pazopanib (Votrient)	VEGF, c-kit, PDGFR	RCC; advanced soft tissue sarcoma chemotherapy treated
Vandetanib	VEGFRs, EGFRs and RET	Unresectable locally advanced or metastatic medullary thyroid cancer
Everolimus (Afinitor)	mTOR	Advanced HER2-negative Breast Cancer, Progressive Neuroendocrine Tumours of Pancreatic Origin (PNET), Subependymal Giant Cell Astrocytoma (SEGA), Advanced RCC; soft tissue sarcoma; renal angiomyolipoma

VEGFR, vascular endothelial growth factor receptor; NSCLC, non-small cell lung cancer; BCR-ABL, fusion of abelson (Abl) tyrosine kinase gen at chromosome 9 and break point cluster (Bcr) gene at chromosome 22; CML, chronic myeloid leukemia; EGFR, epidermal growth factor receptor; EML4-ALK, rearrangement of echinoderm microtubule-associated protein-like 4-anaplastic lymphoma kinase; HER2/neu, one of four membrane proteins in EGFR family; PDGFG, platelet-derived growth factor receptor RET: proto-oncogene, encodes receptor kinase for the neurotrophic factor family; CSF-1R, colony stimulating factor; flt3, encodes receptor tyrosine kinase that regulates hematopoiesis; MAP kinase, family of serine threonine proteins responsible for regulating cellular activities, such as apoptosis; c-kit, tyrosine kinase stem cell factor receptor; SMO, smoothened, e transmembrane protein involved in Hodgebog signal transduction; mTOR, mammalian target of rapamycin inhibitor; BRAF, gene encoding for B-Raf, member of raf kinase family.
